# Profile of CO_2_, CO, and H_2_ Emissions from Thermal Oxidation of Polish Coals

**DOI:** 10.3390/ma13040848

**Published:** 2020-02-13

**Authors:** Karolina Wojtacha-Rychter, Adam Smoliński

**Affiliations:** 1 Department of Mining Aerology, Central Mining Institute, Pl. Gwarków 1, 40-166 Katowice, Poland; kwojtacha@gig.eu; 2 Central Mining Institute, Pl. Gwarków 1, 40-166 Katowice, Poland

**Keywords:** carbon oxides, hydrogen, endogenous fire, oxidation, coal, HCA analysis

## Abstract

The self-heating phenomenon of coal leads to work safety hazards in underground mining. The quantitative analysis of gaseous products in mine atmosphere constitutes one of the detection methods of advanced coal heating. The article presents the results of tests on CO, CO_2_, and H_2_ emissions during simulated heating of coal in the temperature range of 323–523 K. The oxidation of 15 Polish coals of various carbon contents was performed using a flow reactor technique. A chromatography method was applied to measure the changes of oxidation products concentrations with the increase of temperature. It has been determined that all the tested gases were generated at the initial temperature. The collected data indicated that CO_2_ was a major oxidation product in the entire temperature range, while the amounts of H_2_ produced did not exceed 0.49% volume. At the temperature of 323 K, the ratio of CO_2_/CO was in the range of 10–23 but along with the temperature increase the ratio range narrowed to 3–4. In this paper, a comparison of the physical-chemical properties of the tested coals and the emissions profile of the gases using, among others, the hierarchical clustering analysis showed that samples with higher oxygen, sulfur, and inertinite content as well as lower ash and carbon content formed larger amounts of fire gases.

## 1. Introduction

Endogenous fires constitute the largest safety problem and challenge faced by the coal industry. The fires occur all over the world, mostly in major coal-producing countries—such as China, India, Russia, the United States, Australia, and South Africa—but also in a smaller scale in Germany, Poland, or the Czech Republic [1]. For the period of 2014–2018, endogenous fires were some of the most frequent natural hazards occurring in Polish coal mines; 60% out of the total of 59 reported underground mine fires were caused by endogenous fires [2]. The coal self-heating process occurring in the range from the primary temperature of the coal to the critical temperature is one of the decisive factors of endogenous fire risk in coal mines because above the critical temperature the heating rate significantly and dynamically increases. The self-ignition process takes place as a result of coal’s ability to accumulate the heat emitted from the exothermic reaction of coal with oxygen from the ventilation air or naturally accumulated within a coal mass. The loosening of the coal resulting from mining activities or tectonic motions and porous structure of coal enables the transport of oxygen through coal mass and its adsorption on the exposed surface. As a result of coal oxidation, less or more stable coal-oxygen complexes are formed. Along with the increase of temperature, these less stable coal–oxygen complexes are decomposed giving mainly gaseous products like CO, CO_2_, and water vapor [3]. In reality, the normal mine air consists mainly of O_2_, N_2_, and CO_2_ in the amount of approx. 20.5 vol %, 79.5 vol %, and 0.05 vol %, respectively [4]. Carbon monoxide and hydrocarbons can also be present in mine air but at the level of only a few parts per million. Any deviation of the proportion of all these gases in mine atmosphere, namely the increase of the major (carbon monoxide and carbon dioxide) and minor (hydrogen, ethylene, propylene, and acetylene) combustion products may indicate fire development. Hence, the process is mainly detected by monitoring the changes of mine air composition and coal temperature using thermal devices.

A number of studies have been conducted on the carbon dioxide and carbon monoxide emissions during the spontaneous heating of coal. Baris et al. [5] analyzed the quantities of carbon monoxide and carbon dioxide produced from different rank Turkish coals oxidized at the temperatures of 313, 333, and 363 K. It was found that carbon oxides production rate has been positively correlated with temperature and does not depend on particle size of coal. A regular increase of carbon monoxide concentration with the temperature rise was also observed in the work by Lu et al. [6], while the impact of macerals on spontaneous heating liability of coal-shale was studied in the work by Onifade et al. [7]. Yuan and Smith [8] studied the formation of carbon oxides from spontaneous heating of the U.S. coals using an isothermal oven under different airflow ventilation rates and noticed that the CO/CO_2_ ratio is independent from airflow rates at the low initial temperature, but it decreases below 0.2 with the increase of temperature. The experimental findings of this work indicate also that carbon monoxide was detected earlier than carbon dioxide. The studies in works by Wang et al. [9,10] or Wojtacha-Rychter and Smoliński [11] indicate that carbon dioxide emission is larger than that of carbon monoxide. In papers [3,12], the authors determined the activation energy for the production of carbon dioxide and carbon monoxide indicating various values of energy, and discovered that the activation energy for the production of CO_2_ is greater than for CO. Additionally, the study by Zhang et al. [13] showed that the activation energy of carbon monoxide generation increases with the increase of the metamorphism degree of coal. In general, it is also recognized that the decomposition of carbonyl groups leads to the emission of carbon dioxide, while the decomposition of carboxyl groups leads to the formation of carbon monoxide [14,15]. A detailed review of knowledge on the coal oxidation process at low temperature has been presented in the work by Wang at el. [16].

Few studies on the hydrogen detection during spontaneous heating of coal were performed [17,18,19], although it is a gas widely used as one of the indicators of fire hazard, e.g., in Australia [20,21], the U.S. [22], or Poland [23]. It was observed that hydrogen can be detected at the temperature of 328–368 K. However, a significant amount of hydrogen is mainly produced at the stage of advanced heating. Hydrogen may be formed in various ways such as the dehydrogenation of alkanes to alkenes, in the reaction of carbon monoxide or methane with water vapor [24] as well as the decomposition of formaldehyde [25,26,27].

The objective of the study was to investigate the influence of temperature and coal types on the amount of carbon monoxide (CO), carbon dioxide (CO_2_), and hydrogen (H_2_) released during the thermal oxidation of Polish coals in the temperature of 323, 373, 423, 473, and 523 K. In this paper, a chemometric method of hierarchical clustering analysis (HCA) was applied to investigate the correlation between coal parameters and gases concentrations at the outlet of the reactor. The findings of this study improve the knowledge in the field of the relationship between coal properties and carbon monoxide, carbon dioxide, and hydrogen emissions; in addition, they can find practical implications for work safety in the coal mining industry.

## 2. Materials and Methods

### 2.1. Measurement Method

**Figure 1:**
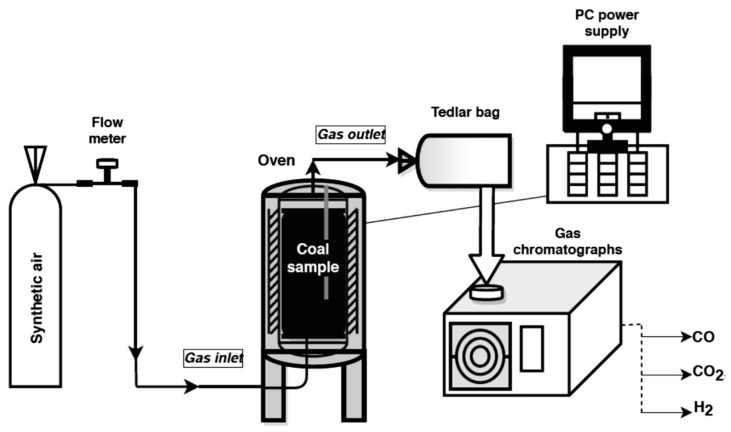
The research stand.

Studies on the emission of carbon dioxide, carbon monoxide and hydrogen from coals oxidized at various temperatures were performed with the application of a specially designed installation located in the Laboratory of Coal Spontaneous Combustion of Central Mining Institute, Poland. The experiment stand for simulating the coal self-heating process is shown in Figure 1.

The main element of the test stand is a 0.7 L reactor made of stainless steel with an inlet of air stream and an outlet of the gaseous products. The reactor is placed in the center of a resistance oven which carries out the tests in the temperature range from 323 K to 573 K. The interior of the cylinder-shaped furnace is heated using an automatic switch-off. Two thermocouples allow monitoring the temperature inside the crushed coal and in the furnace. The signals from both temperature sensors are monitored by computer software.

During the experiment, a coal sample of a grain size below 2 mm and weight of 0.4 kg was placed on a steel mesh located 1 cm above the bottom of the reactor for better distribution of the synthetic air flow. The air from a gas cylinder was injected to the bottom of the reactor at the flow of 5 × 10^−7^ m^3^/s and passed through the sample. ALICAT Scientific MS–Series Mass Flow Meter (Alicat Scientific, Tucson, AZ, USA) was applied to monitor the value of the flow rate. Simultaneously, a crushed coal sample packed in the center of reactor was heated to the set temperature. The first gaseous mixture was collected at the temperature of 323 K. The tests were conducted up to the temperature of 523 K. Samples of the released gases were taken manually to an airtight Tedlar bag for the analyses. Carbon dioxide, carbon monoxide, and hydrogen concentrations in the multicomponent gaseous mixture in the outflow of the reactor and at the set temperature were measured with the application of gas chromatographs (3000A, Agilent, Santa Clara, CA, USA). A SRI 8610C chromatograph (SRI Instruments, Earl St. Torrance, CA, USA) was used for the quantitative analysis of carbon dioxide. The chromatograph is equipped with two packed columns, one of which is filled with a molecular sieve 5A and the other with Porapak Q material, whereas hydrogen content was measured with the application of Shimadzu GC 2014 chromatograph (Shimadzu, Kyoto, Japan). The measurements were performed under isothermal conditions; the temperature of molecular sieve 5A packed column was programmed to 303 K. The above-mentioned chromatographs were equipped with thermal conductivity detectors. A gas chromatograph, model Shimadzu GC 17A, with flame ionization detector and 5A molecular sieve layer inside a capillary column (column temperature 357 K) was used for determining carbon monoxide. Gas samples were introduced in the volume of 120 mL each. In the case of hydrogen, pure argon was selected as a carrier gas. For the remaining gases, pure helium was chosen as a carrier medium. With this setup, the limit of carbon monoxide quantification was of 0.0001 vol %, carbon dioxide around 0.05 vol %, while hydrogen was 0.00005 vol %.

### 2.2. Sample Characterization

The 15 different coals of relatively low (<70% w/w (w/w i.e., weight/weight, mass fraction), medium (70–80% w/w) and high (>80% w/w) carbon content, coming from active mines located in the area of the Upper Silesian Coal Basin (USCB), in the south of Poland, were selected as the tested samples. The coal samples were analyzed in terms of their proximate, ultimate and petrographic parameters according to relevant standards in an accredited laboratory of the Department of Solid Fuel Quality Assessment at the Central Mining Institute and the results in the analytical state are given in Table 1.

Detailed information concerning the analyzers and standards has been reported elsewhere [11,28]. Sample nos. 1–5 have vitrinite reflectance below 0.6%, so according to the United Nations Economic Commission for Europe in-seam coal rank classification [29] they are defined as low rank coals (lignite), while sample nos. 6–15 with vitrinite reflectance above 0.6% are classified as medium rank coal (bituminous). Lignite samples are characterized by low carbon content from 61.54% w/w to 67.10% w/w, as well as the highest oxygen content in the range of 9.26–12.78% w/w.

Sample no. 1 was an exception for which the oxygen content was at the level of 4.79% w/w. This sample is also characterized by the highest ash content (26.98% w/w) as well as the lowest sulfur and moisture contents. The carbon content of five bituminous coal samples nos. 6–10 was in the range of 70–80% w/w, while for sample nos. 11–15 it was higher than 80% w/w. In coal sample nos. 6–15, the oxygen content changed from 8.19% w/w to 1.09% w/w. The lowest oxygen content of sample no. 15 corresponded with the highest carbon content equal to 89.11% w/w. The petrographic analysis of coals showed that coal sample nos. 4, 6, 7, 11, 12, 13, and 15 are characterized by a high content of inertinite maceral in the range from 44 vol % to 58 vol %. The lowest values of inertinite were determined in coal sample nos. 3 and 14 (below 20 vol %).

## 3. Results and Discussion

### 3.1. Gases Emission Profile

**Figure 2:**
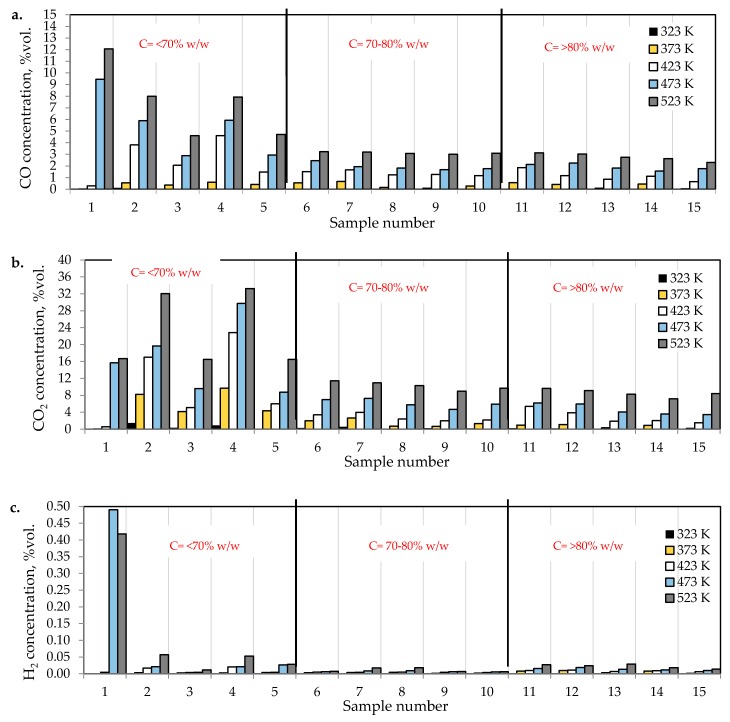
The amounts of (**a**) carbon monoxide, (**b**) carbon dioxide, and (**c**) hydrogen emitted from coal samples oxidized in the temperature range of 323–523 K.

Figure 2a–c presents the changes of carbon monoxide, carbon dioxide and hydrogen % concentrations during the coal heating process under laboratory conditions.

**Figure 3:**
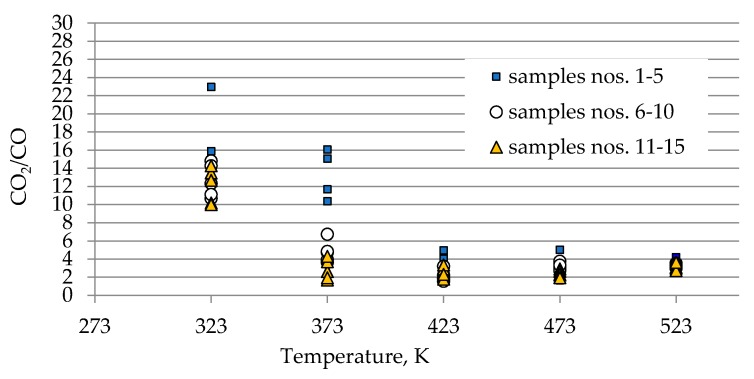
Changes of CO_2_/CO ratio against coal temperature.

As can be seen in Figure 2a–c, the concentration of carbon oxides and hydrogen regularly increased with the increase of coal temperature. The results show that carbon dioxide is the main combustion product in the gaseous mixture at the outlet of the reactor, which is in line with the works by Wand et al. [9] and Baris and Didari [30]. The amount of hydrogen formation did not exceed 0.06 vol %. in the entire temperature range, except for coal sample no. 1 for which the amount of hydrogen released at the temperatures of 473 K and 523 K is of the order of 0.49 vol %. At the temperature of 323 K, the amount of hydrogen production varied from 0.0001 to 0.0018 vol %. The high emission of hydrogen for coal sample nos. 1, 2, and 4 corresponded to the highest emission of carbon monoxide. It can be observed that the production of hydrogen was higher for the highest rank coals (coal sample nos. 11–15) than for the medium rank coals (coal sample nos. 6–11). In the entire temperature range, the amount of carbon dioxide emission was ≥2-fold higher as compared to carbon monoxide. As shown in Figure 2a, in the temperature range of 323–373 K, carbon monoxide was detected at the level of a few hundred parts per million, while with temperature close to 423 K, the carbon monoxide concentrations reached the values of 1–2 vol %. The exceptions were observed for coal sample nos. 2 and 4, for which a carbon monoxide concentration at the temperature of 423 K increased up to the values of 3.81 vol % and 4.61 vol %, respectively. The concentrations of carbon dioxide in the multicomponent mixtures increased to 2–7 vol %. with temperature level of 423 K, while for samples 2 and 4 the gas content was even 17.03 vol % and 22.82 vol %, respectively (see Figure 2b). The lower concentration of carbon monoxide in comparison to carbon dioxide is due to its high activation energy. Wang et al. [3] found that the activation energy for the formation of carbon dioxide is equal to 52.1 ± 6.3 kJ/mol, while energy activation of carbon monoxide to 72.0 ± 5.8 kJ/mol. This means that carbon monoxide requires a larger amount of energy (in the form of heat) to overcome the energy barrier associated with activation energy in comparison to carbon dioxide. Figure 3 presents the ratio of CO_2_ and CO at given temperatures.

At the temperature of 323 K and 373 K, the ratio gained high values in the range of 10–23 and 2–16 respectively, because of low carbon monoxide emission. Along with the coal temperature increase to the values of 423 K and 472 K, the ratio decreased to the much narrower range of 1–5 due to a significant increase in the amount of CO generation. By contrast, at the highest temperature of 523 K, the ratio continues to be stable and the value is in the range of 3–4, independently of coal rank used. This implies that at the beginning of the coal spontaneous heating the ratio of CO_2_/CO can reach high values, in particular for low rank coal, and, as a result of a further thermal runaway this ratio will tend to decrease to a constant value. In previous works [3,10,31], the decreasing trend of CO_2_/CO ratio with the increase of coal temperature was also observed.

Based on the results, it was found that carbon dioxide is released in abundant quantities (at a level of several percent) already at low temperatures compared to carbon monoxide, which may suggest that this gas is a better indicator of fire risk. However, the use of carbon dioxide as a fire detecting gas has some limitations, making this gas unreliable. The gas can originate from sources other than the self-heating process, i.e., natural content, acid mine water neutralization, machines with diesel-fueled engines working underground or the injection of carbon dioxide into a sealed area as an inert gas [24,32]. Additionally, studies on the fire gases sorption on coal also showed that carbon dioxide is easily absorbed on coal surface due to its small kinetic diameter (0.33 mm), a linear structure and a high quadruple moment of molecules [28,33,34]. This phenomenon may result in underestimating the value of the gas concentration at the monitoring station, especially when the distance between the source of fire and the sampling point is long. Besides, the higher density of this gas means that it will flow near the floor, especially when the velocity of atmospheric air supplied to the mine (ventilation) is low. The limitations indicate the importance of carbon monoxide over carbon dioxide. However, as the experimental results show, it is required to use an analytic method with the detection limits at the level of 1 part per million to enable the quantification of carbon monoxide at the lower temperatures.

### 3.2. Influence of Coal Properties on the Amount of CO_2_, CO, and H_2_ Produced

#### 3.2.1. Carbon Content

The results presented in Figure 2a–c and Figure 3 also show a dependence of the amounts of gases emitted on the coal properties. In Figure 3 it was found that at the temperature of 323 K and 373 K, the highest values of the ratio of CO_2_/CO were observed mostly for samples with the low carbon content. These findings may suggest that the ratio can depend on coal rank, particularly in the initial stage of coal self-heating process. As can be seen in Figure 2a–c, the maximum concentrations of carbon oxides and hydrogen at the outlet of the reactor were observed for coal sample nos. 1–6 characterized by the low carbon content, below 70 vol %. For three lignite samples of numbers 1, 3, and 5 at the highest temperature of 523 K, the carbon dioxide concentration was approx. two times higher than for bituminous samples, and it was equal to 16.70 vol %, 16.49 vol % and 16.50 vol %, respectively, as presented in Figure 2b. By contrast, the concentrations of carbon monoxide were only 1.5–2 times higher, except for coal sample no. 1, for which the CO content was 4-fold higher. For coal sample nos. 2 and 4, the concentrations of carbon dioxide in this temperature were even 4-fold higher than for bituminous samples; however, the concentrations of carbon monoxide were even 3–4 times higher than for coal sample nos. 6–15. Coal sample nos. 2 and 4 gained the concentration of carbon dioxide at the level of 8–9 vol %. at the much lower temperature of 373 K. For sample nos. 6–10 the high concentrations of CO_2_ and CO were noted at the temperature of 523 K, these values were in the following ranges of 8.99–11.41 vol % and 3.00–3.23 vol % respectively. For the third group of coal sample nos. 11–15 and at the same temperature of 523 K, the concentration of carbon dioxide changed from 7.20 to 9.64 vol %, while the content of carbon monoxide was in the range of 2.31–3.13 vol %, so the values were even 20–30% lower than for coal sample nos. 6–10. A high amount of carbon oxides produced from low rank coal can be explained by the loosely packed structure of these samples. The condensed homo- or heterocyclic aromatic compounds constitute the basic structural unit of coal; the structures are connected by means of extreme groups consisting of aliphatic and alicyclic chains [35]. Within the groups, the carbon and hydrogen atoms are partly replaced by oxygen atoms, forming various functional groups (i.e., hydroxyls –OH, methoxyls –OCH_3_, carboxyls –COOH, or carbonyls =CO) on the coal surface. Thus, the high oxygen content from ultimate analysis may suggest a high number of active oxygen containing functional groups. As the coalification process progresses, the degree of ordering and the share of aromatic compounds increase, whereas the number of side groups decreases [36,37]. Aromatic structural units are more packed, which makes that the flow of oxygen deep inside the coal structure is more difficult. Besides, a larger amount of carbon monoxide and carbon dioxide generated from coal sample nos. 2–5 with high oxygen content (above 9% w/w) may also originate from the decomposition of the active oxygen containing functional groups (i.e., hydroxyls, carboxyls, carbonyls or aldehydes) located in the coal matrix [13,14].

#### 3.2.2. Oxygen Content

**Figure 4:**
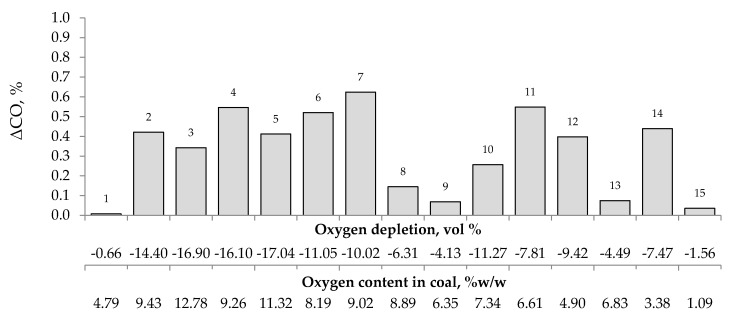
The relationship between the growth of carbon monoxide concentration in the temperature range of 323–373 K and oxygen content in coals as well as oxygen depletion in the gaseous mixture.

**Figure 5:**
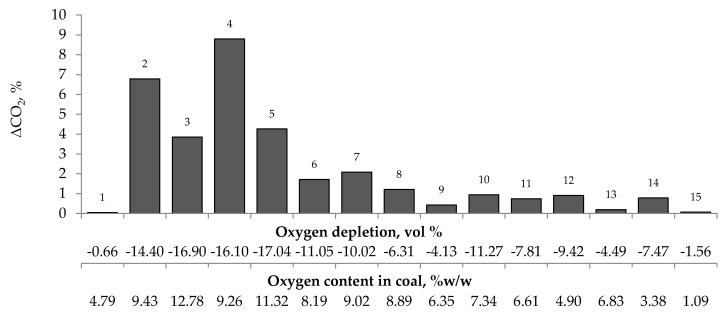
Relationship between the growth of carbon dioxide concentration in the temperature range of 323–373 K and oxygen content in coals as well as oxygen depletion in the gaseous mixture.

**Figure 6:**
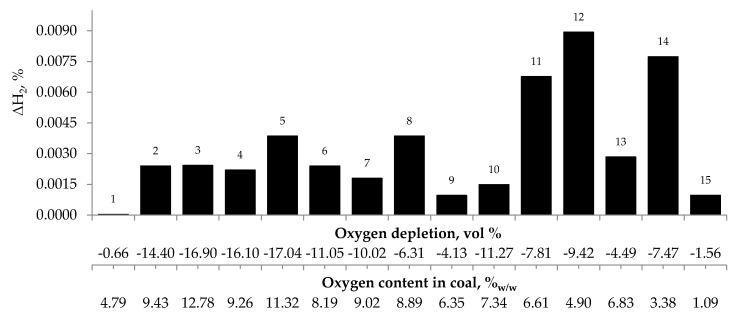
Relationship between the growth of hydrogen concentration in the temperature range of 323–373 K and oxygen content in coals as well as oxygen depletion in the gaseous mixture.

Figure 4, Figure 5 and Figure 6 present the growth of carbon monoxide (ΔCO), carbon dioxide (ΔCO_2_) and hydrogen (ΔH_2_) emission in the temperature range of 323–373 K, respectively as a function of oxygen content in coal and oxygen depletion in the gaseous mixture at the outlet of the reactor. As can be seen in Figure 4 and Figure 5, the large growth of carbon oxides corresponds to high oxygen depletion during the coal oxidation reaction. For example (see Figure 4), for coal sample nos. 1, 8, 9, 13, and 15, the growth of carbon monoxide was below 0.15 vol % and the oxygen depletion did not exceed 6.31 vol %, while for the remaining samples the gas growth was over 0.34 vol % and the depletion of oxygen was over 7.81 vol %. Moreover, it was found that for coal sample nos. 2–7 with oxygen content in coal above 8% w/w the values of ΔCO_2_ (see Figure 5) were in the range of 1.21–8.79 vol %, in addition, they were higher than for samples with lower oxygen content.

These findings suggest that at the beginning of the spontaneous heating, the CO emission originates mainly from the decomposition of the intermediate oxygenated complexes formed during the coal oxidation reaction, while the carbon dioxide emission may also come from oxygen containing groups placed in coal structure. No correlation between oxygen depletion and oxygen content in coal and the amount of emitted hydrogen was found (see Figure 6).

#### 3.2.3. Ash and Sulfur Content

**Figure 7:**
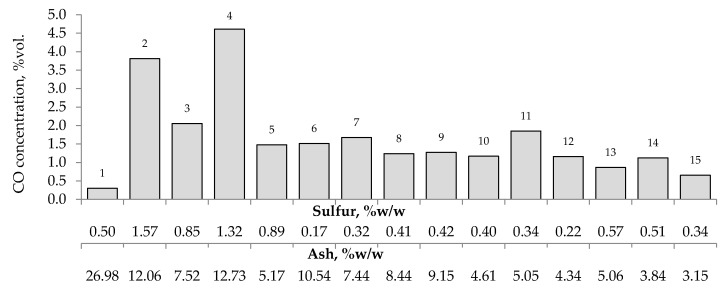
Relationship between the amount of carbon monoxide produced from samples oxidized at the temperature of 423 K versus sulfur and ash content in coal.

**Figure 8:**
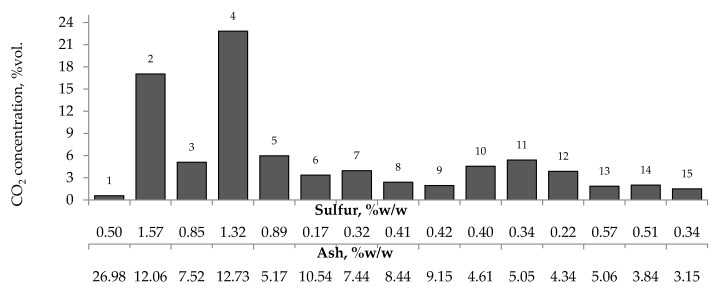
Relationship between the amount of carbon dioxide produced from samples oxidized at the temperature of 423 K versus sulfur and ash content in coal.

**Figure 9:**
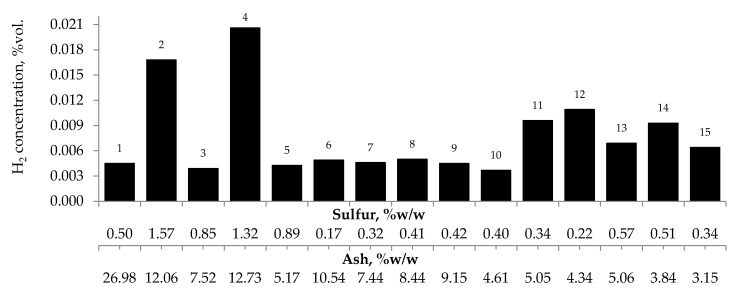
Relationship between the amount of hydrogen produced from samples oxidized at the temperature of 423 K versus sulfur and ash content in coal.

Figure 7, Figure 8 and Figure 9 present the relationship between the amounts of carbon oxides and hydrogen generated during coal oxidation at the temperature of 423 K and sulfur content as well as ash content in coal. The results show that a low oxidation rate and formation of the small amount of gaseous products from coal sample no. 1 can be caused by the high ash content (26.98% w/w), whereas a high sulfur content increases the susceptibility of coal sample nos. 2 and 4 to spontaneous combustion. It is commonly known that some of the minerals within coal, for example pyrite may accelerate the self-heating process [38,39,40]. The exothermic reaction of pyrite oxidation due to the interaction of water and oxygen produces heat, which under favorable conditions leads to coal self-heating. The enthalpy value of the reaction almost twice as high as for coal and the low specific heat value of pyrite cause an increase in pyrite temperature almost three times higher than for coal. In addition, the pyrite oxidation products have a larger volume in relation to the substrates, which causes the increase of pyrite volume and the formation of cracks, and thus an easier oxygen flow to the reaction zone. Based on the above information, it can be explained that a high content of sulfur (above 1% w/w) in coal sample nos. 2 and 4 may contribute to the higher oxidation rate and high gases emission of these coals as compared to the other samples.

#### 3.2.4. Maceral Content

**Figure 10:**
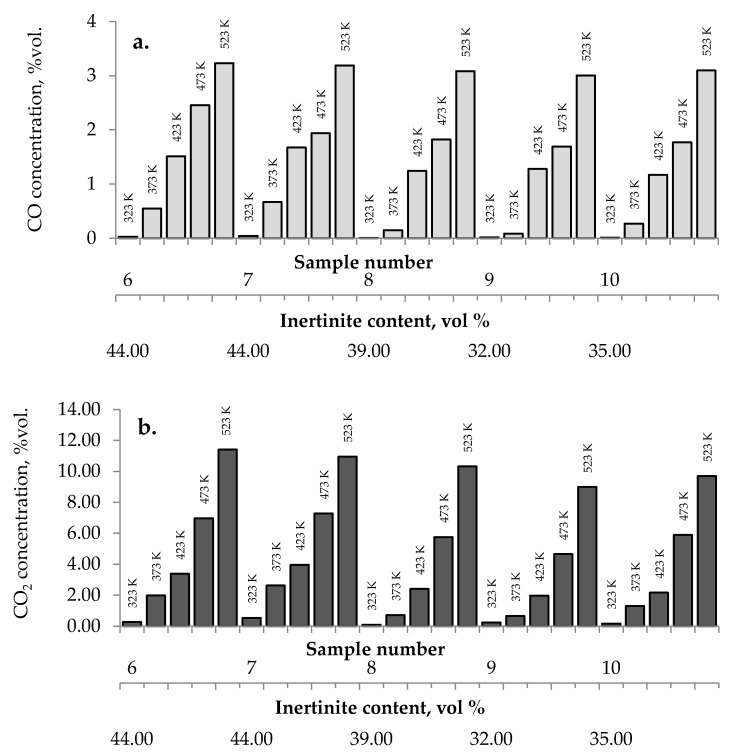

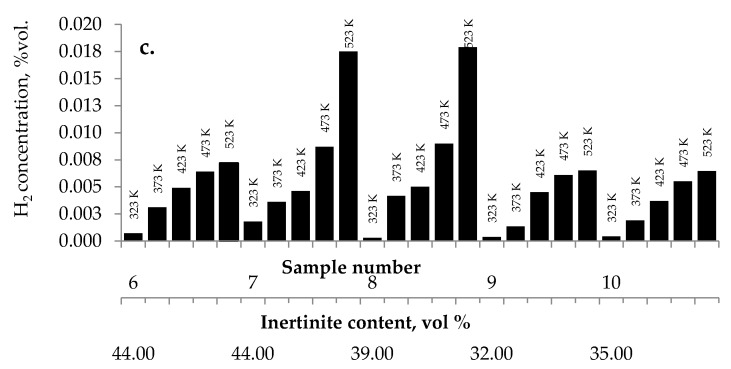
Relationship between the amount of (**a**) carbon monoxide, (**b**) carbon dioxide, and (**c**) hydrogen released from coal sample nos. 6–10 and inertinite content in coal.

No influence of maceral content on the amount of carbon dioxides and hydrogen emission from the highest rank coals was observed. The impact of inertinite content became clearer for samples with carbon content in the range of 60–80% w/w. As can be seen in Figure 2a–c and Figure 10, the amounts of carbon oxides produced from coal sample nos. 6, 7, and 4 are found to be higher than those for coal sample nos. 1–3, 5, 8–10.

Coal sample nos. 6, 7, and 4 were characterized by the highest inertinite content: 44 vol %, 44 vol %, and 51 vol %, respectively, while the inertinite content for the remaining seven samples was in the range of 22–39 vol % This is in agreement with the results of works [5] demonstrating that samples with a high amount of inertinite macerals have the highest production rate of carbon monoxide and carbon dioxide. In work by Kaymakçi and Didari [41], a high correlation between crossing point temperature and inertinite content was found. Macerals from the inertinite group are characterized by greater porosity than macerals from the vitrinite group with a high content of meso- and macropores [42,43,44]. The macroporosity of the petrographic component of the inertinite group means that they play significant role as transport channels for the flowing gas, and thus allow oxygen to flow more easily to the internal coal surface. Besides, the amount of oxygen containing functional groups such as carbonyl and carboxyl in inertinite is higher than in vitrinite [45]. No relationship between the inertinite content and the amount of emitted hydrogen was noticed.

### 3.3. Hierarchical Clustering Analysis

**Figure 11:**
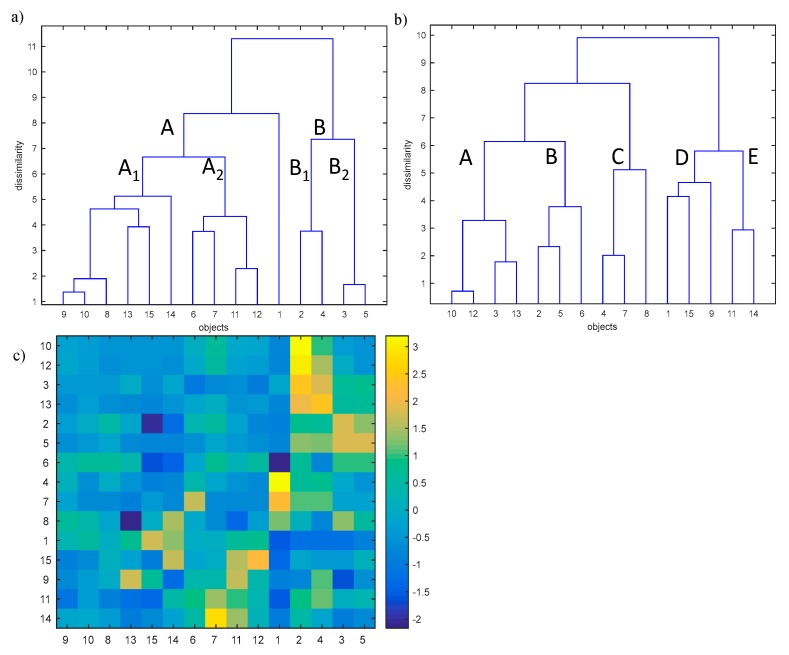
Dendrograms for (**a**) objects (15 studied coal samples with low, medium and high carbon content in the space of 15 measured parameters, and (**b**) the parameters in the object space with (**c**) the colour map showing the values of measured parameters for the particular samples.

The analysis of the complex effect of the physical and chemical parameters on the self-heating of coal was performed with the application of hierarchical clustering analysis (HCA) [46,47,48,49,50]. HCA allows to reveal the internal data structure (clustering tendency) through investigating the similarities of coal samples (objects) in the space described by studied parameters or similarities between parameters in the space described by studied objects. The analysis is characterized by the similarity measure (Euclidean or Manhattan distances) used as well as by the way how the sub-clusters are linked (i.e., single linkage, complete linkage, centroid linkage, average linkage, and Ward linkage). In our study, the Ward linkage method with the Euclidean distance measure was used. In order to apply the HCA, the studied data were organized in the matrix X (15 × 15). The rows of the matrix X represent the studied coal samples of relatively low (<70% w/w, objects nos. 1–5), medium (70–80% w/w, objects nos. 6–10) and high (>80% w/w, objects nos. 11–15) carbon content. The columns of the matrix X represent carbon, oxygen, sulfur, ash, moisture, volatile matter, mineral matter, vitrinite, and inertinite content in the studied samples (parameters nos. 1–9) and the amount of gases (CO, CO_2_, and H_2_) emitted during the self-ignition process at the temperature of 323 and 373 K, respectively (parameters nos. 10–15). The HCA allows to effectively investigate the similarity between studied coal samples in the parameters space and the similarities between studied parameters in the objects space. The HCA dendrograms were developed with the use of Ward’s linkage method and the Euclidean distance. The studied dendrograms are presented in Figure 11.

Dendrograms presented in Figure 11a,b allow to investigate the similarities between objects and parameters separately but do not allow the simultaneous interpretation of the observed phenomenon. To address this disadvantage, an additional graph was added (see Figure 11c) which represents the values of studied parameters in the form of color bars. The color bars were sorted according to the order observed on dendrograms presented in Figure 11a,b, respectively.

The dendrogram presented in Figure 11a allows to group studied coal samples into two clusters A and B. Additionally, the uniqueness of coal sample no. 1 was also observed. Cluster A collects coal sample nos. 6–15 whereas cluster B is composed of coal sample nos. 2–5. Moreover, within cluster A, a sub-cluster A1 including coal sample nos. 8–10, 13–15, as well as the sub-cluster A2 including studied coal sample nos. 6, 7, 11, and 12 were distinguished. In the similar way, within cluster B the following two sub-clusters can be observed:

—Subcluster B1 composed of coal sample nos. 2 and 4, and

—Subcluster B2 collecting the coal sample nos. 3 and 5, respectively.

The dendrogram constructed for studied parameters in the objects space (see Figure 11b) allows to classify the parameters into five classes:

—Cass A containing parameters nos. 10 and 12 (amount of CO and CO_2_ emitted at the temperature of 323 K),

—Class B composed of parameters nos. 2, 5, and 6 (oxygen, moisture and volatile matter content in the coal sample),

—Class C collecting parameters nos. 4, 7, and 8 (ash, mineral matter and vitrinite content in the coal sample), 

—Class D grouping parameters nos. 1, 9, and 15 (carbon and inertinite content in the coal sample and the amount of H_2_ emitted at the temperature 373 K), and

—Class E containing parameters nos. 11 and 14 (amount of CO and H_2_ emitted at the temperature of 373 K and 323 K, respectively).

In order to explain the reason of grouping the studied samples into two main clusters as well as to explain the uniqueness of coal sample no.1, the dendrogram of objects in the space of studied parameters with the color map of studied standardized data (see Figure 11c) sorted according to the order of objects and parameters on the dendrograms (see Figure 11a,b) was applied. Based on that, a conclusion may be drawn that all coal samples of medium (objects nos. 6–10) and high (objects nos. 11–15) carbon content belonging to cluster A are characterized by a relatively low sulfur content in the sample and a low amount of CO and CO_2_ emitted at the temperature of 323 K and 373 K, and, additionally, the low amount of H_2_ emitted at the temperature of 323 K (parameters nos. 3 and 10–14) among all studied coal samples. Sub-cluster A1 collects coal samples which are characterized by a relatively higher carbon content in the sample (parameter no. 1) among all studied coal samples. Moreover, coal sample no. 13 is unique due to the highest inertinite content in the sample (parameter no. 9) and the lowest vitrinite content in the sample (parameter no. 8), whereas the coal sample no. 15 is characterized by the highest content of carbon and the lowest content of oxygen in the sample (parameters nos. 1 and 2). By contrast, coal sample no. 14 is characterized by the high amount of H_2_ emitted at the temperature of 373 K (parameter no. 15) and the highest vitrinite content in the sample (parameter no. 8).

The objects belonging to sub-cluster A2 are described by a relatively higher inertinite content in the sample as well as a higher amount of CO and H_2_ emitted at the temperature of 373 K and 323 K (parameters nos. 9, 11, and 14). Moreover, coal sample nos. 6 and 7 are characterized by the highest volatile and mineral matter contents in the coal samples (parameter nos. 6 and 7), respectively, whereas object no. 12 is unique due to the highest amount of H_2_ emitted at the temperature of 373 K (parameter no. 15).

Non-clustered coal sample no. 1 is unique due to the lowest volatile matter content (parameter no. 6) and the highest ash and mineral matter content in the sample (parameters nos. 4 and 7).

Cluster B collecting the remaining coal samples with low carbon content (objects nos. 2–5, parameter no. 1) is characterized by higher content of oxygen, sulfur, moisture, and the amount of CO_2_ emitted at the temperature of 373 K (parameters nos. 2, 3, 5, and 13). The sub-cluster B1, collecting coal sample nos. 2 and 4, is additionally unique due to the higher amount of CO and CO_2_ emitted at the temperature of 323 K (parameter nos. 10 and 12). Moreover, coal sample no. 2 is characterized by the highest sulfur content in the sample and the highest amount of CO and CO_2_ emitted at the temperature of 323 K (parameter nos. 3, 10, and 12) among all studied coal samples, whereas coal sample no. 2 is characterized by the highest amount of CO_2_ emitted at the temperature of 373 K (parameter no. 13). 

Sub-cluster B2 is unique due to the highest moisture content in the coal sample (parameter no. 5). Additionally, coal sample no. 3 is characterized by the highest oxygen content in the sample (parameter no. 2).

## 4. Conclusions

The results presented in this work provide evidence that carbon dioxide, carbon monoxide and hydrogen are sensitive to coal heating temperature and their concentration increases regularly with the temperature of coal. The emission of carbon dioxide is much higher than that of carbon monoxide probably as a result of the lower value of activation energy. It was noted that at the beginning of the test, the CO_2_/CO ratio was above 10 and then it showed a decreasing tendency to the level of constant value when a controlled rise of temperature occurred. Based on the measurements, it can be concluded that coals even of the same rank may exhibit significant differences in the amounts of gaseous products generated during the thermal oxidation process due to their various physical and chemical properties. The differences in the tendency to the spontaneous heating of coals were mainly caused by the different carbon, oxygen, sulfur, and ash as well as inertinite contents in the studied coal samples. The lignite oxidation process was characterized by a higher total amount of carbon oxides generated in comparison to the values obtained for the bituminous samples. High sulfur content in lignite and high inertinite content in bituminous contributed to a significantly higher emission of carbon dioxide and carbon monoxide. The chemometric method such as HCA enabled to effectively explore the relationship between the amounts of carbon monoxide, carbon dioxide, and hydrogen generated during the oxidation process and the properties of coal. In the research, no correlation between coal parameters and the production of hydrogen was observed. In the case of the low temperature oxidation process, hydrogen reached concentration values at the level of a few parts per million. This means that the high concentration of hydrogen in underground mine atmosphere could result in an advanced heating phase.

## Figures and Tables

**Table 1:** Proximate and ultimate analysis of coal samples tested.

No	Ultimate Analysis					Proximate Analysis				Petrographic Analysis			
	C_a_^1^	H_a_^2^	O_a_^3^	N_a_^4^	S_a_^5^	A_a_^6^	W_a_^7^	V_a_^8^	MM_a_^9^	R_o a_^10^	V_a_^11^	L_a_^12^	I_a_^13^
1	61.54	3.66	4.79	1.04	0.50	26.98	1.11	17.65	16	0.59	69	1	30
2	63.98	4.12	9.43	0.89	1.57	12.06	8.08	29.2	10	0.55	55	14	31
3	64.18	4.13	12.78	0.90	0.85	7.52	9.69	30.55	3	0.52	70	8	22
4	64.37	3.68	9.26	0.85	1.32	12.73	7.84	23.38	10	0.51	44	5	51
5	67.10	3.92	11.32	1.07	0.89	5.17	9.68	30.66	3	0.57	61	6	33
6	74.16	4.18	8.19	1.23	0.17	10.54	1.53	25.62	13	0.93	52	4	44
7	74.66	4.16	9.02	1.41	0.32	7.44	3.02	29.76	1	0.76	45	11	44
8	75.17	4.22	8.89	1.41	0.41	8.44	3.02	29.25	1	0.83	51	5	39
9	76.89	4.31	6.35	1.36	0.42	9.15	1.67	28.12	3	0.91	62	6	32
10	79.59	4.68	7.34	1.48	0.40	4.61	2.15	29.29	1	0.88	59	7	35
11	80.10	4.66	6.61	1.33	0.34	5.05	2.11	27.65	1	0.87	36	7	57
12	80.65	4.90	4.90	1.45	0.22	4.34	1.70	29.05	1	0.95	49	7	44
13	81.01	4.26	6.83	1.32	0.57	5.06	1.34	28.33	0	0.88	26	16	58
14	85.42	4.42	3.38	1.46	0.51	3.84	0.99	20.38	1	1.15	72	2	25
15	89.11	4.16	1.09	1.39	0.34	3.15	0.93	19.68	2	1.28	53	1	46

Note: ^1^carbon (%w/w), ^2^hydrogen (%w/w), ^3^oxygen (%w/w), ^4^nitrogen (%w/w), ^5^sulfur (%w/w), ^6^ash (%w/w), ^7^moisture (%w/w), ^8^volatile matter (%w/w), ^9^mineral matter (vol %), ^10^vitrinite reflectance (vol %), ^11^vitrinite (vol %), ^12^liptinite (vol %), ^13^inertinite (vol %). Subscript: a—analytical state.
